# Radiosensitivity in breast cancer assessed by the Comet and micronucleus assays

**DOI:** 10.1038/sj.bjc.6603005

**Published:** 2006-03-14

**Authors:** C S Djuzenova, B Mühl, M Fehn, U Oppitz, B Müller, M Flentje

**Affiliations:** 1 Klinik für Strahlentherapie der Universität Würzburg, Josef-Schneider-Str. 11, Würzburg D-97080, Germany

**Keywords:** DNA damage, DNA repair, peripheral blood lymphocytes, single-cell gel electrophoresis

## Abstract

Spontaneous and radiation-induced genetic instability of peripheral blood mononuclear cells derived from unselected breast cancer (BC) patients (*n*=50) was examined using the single-cell gel electrophoresis (Comet) assay and a modified G2 micronucleus (MN) test. Cells from apparently healthy donors (*n*=16) and from cancer patients (*n*=9) with an adverse early skin reaction to radiotherapy (RT) served as references. Nonirradiated cells from the three tested groups exhibited similar baseline levels of DNA fragmentation assessed by the Comet assay. Likewise, the Comet analysis of *in vitro* irradiated (5 Gy) cells did not reveal any significant differences among the three groups with respect to the initial and residual DNA fragmentation, as well as the DNA repair kinetics. The G2 MN test showed that cells from cancer patients with an adverse skin reaction to RT displayed increased frequencies of both spontaneous and radiation-induced MN compared to healthy control or the group of unselected BC patients. Two patients from the latter group developed an increased early skin reaction to RT, which was associated with an increased initial DNA fragmentation *in vitro* only in one of them. Cells from the other BC patient exhibited a striking slope in the dose–response curve detected by the G2 MN test. We also found that previous RT strongly increased both spontaneous and *in vitro* radiation-induced MN levels, and to a lesser extent, the radiation-induced DNA damage assessed by the Comet assay. These data suggest that clinical radiation may provoke genetic instability and/or induce persistent DNA damage in normal cells of cancer patients, thus leading to increased levels of MN induction and DNA fragmentation after irradiation *in vitro*. Therefore, care has to be taken when blood samples collected postradiotherapeutically are used to assess the radiosensitivity of cancer patients.

Breast cancer (BC) is the common type of malignancy in females, accounting for approximately 21% of all cancer cases in women worldwide (Parkin *et al*, 1999). Out of all BC patients, 2% have a strong genetic predisposition, caused by the highly penetrant *BRCA1* and *BRCA2* genes (Peto *et al*, 1999). Because these genes cannot account for the overall increased risk in the relatives of BC cases (Baeyens *et al*, 2002), it was suggested that a substantial proportion of BC patients may be predisposed to cancer through mutations in low penetrance genes (Teare *et al*, 1994; Roberts *et al*, 1999; Scott *et al*, 1999; Scott, 2004), which may be genes involved in DNA damage processing and repair.

Several DNA damage processing and repair pathways constitute a guard system that protects cells against genetic instability and tumorigenesis. Both genetic instability and impaired DNA restitution have been pointed out as factors underlying increased susceptibility to malignancy (for reviews, see Lengauer *et al*, 1998; Thompson and Schild, 2002). The biological importance of genetic instability and DNA repair mechanisms in cancer development is particularly well illustrated by the autosomal recessive disorders ataxia telangiectasia, Fanconi anaemia and Nijmegen breakage syndrome. These chromosome breakage syndromes are characterised by various defects in DNA repair, predisposition to different forms of malignancy and increased radiosensitivity (for a review, see Carney 1999). Apart from these rare syndromes, the deficient DNA repair capacity has been proposed to be a predisposing factor in familial BC and in some sporadic BC cases (Parshad *et al*, 1996). Genomic instability has also been described for various hereditary cancers including hereditary BC (Rothfuß *et al*, 2000; Baeyens *et al*, 2002).

Genomic instability and DNA repair capacity have been analysed in numerous population-based studies using a variety of assays that assess chromosomal aberrations, sister chromatid exchanges, micronuclei (MN), DNA fragmentation by means of the Comet assay, etc. Some of these studies have revealed reduced DNA repair capacity in peripheral blood mononuclear cells (PBMCs, exposed *in vitro* to ionising radiation (IR) or UV) from BC patients, as evaluated by the chromosome aberration assay (Rigaud *et al*, 1990; Helzlsouer *et al*, 1995; Parshad *et al*, 1996) as well as by the MN test (Scott *et al*, 1998, 1999; Baeyens *et al*, 2002). Furthermore, a series of studies have found elevated G2 chromosomal radiosensitivity in the blood cells from BC patients (Baria *et al*, 2001; Riches *et al*, 2001; Baeyens *et al*, 2002). Interestingly, there has been no correlation between the G2 chromosome aberration and G0 MN assays performed simultaneously on cells from 80 patients, which were either G2 (38%) or G0 (21%) sensitive, with only 4% sensitive in both assays (Scott *et al*, 1999). Prompted by the finding that the G2 chromosome aberration test yields a larger portion of sensitive patients than the G0 MN test (Scott *et al*, 1999) and by the potency of the G2 metaphase test in assessing cytogenetic responses (Parshad *et al*, 1996; Baria *et al*, 2001; Riches *et al*, 2001; Baeyens *et al*, 2002), we applied here a modified G2 MN test. Unlike the conventional G0 MN test, our method involved cells stimulated with phytohemagglutinin (PHA) for 48 h *prior* to irradiation, as commonly used for assessing G2 chromosomal aberrations (Scott *et al*, 1998).

Another convenient test to evaluate both genetic instability and DNA repair capacity is the single-cell gel electrophoresis or Comet assay (Singh *et al*, 1988; Olive *et al*, 1990). We have shown recently that this method allows the discrimination of carriers of chromosome instability syndromes including ataxia telangiectasia and Fanconi anaemia (Djuzenova *et al*, 1999, 2001). In addition, using the Comet assay, we have found the background and induced DNA damage in the peripheral blood lymphocytes from BC patients to be similar to that in control individuals (Djuzenova *et al*, 1999). Consistent with these data, nonirradiated lymphocytes from patients with multiple tumours (Müller-Vogt *et al*, 2003), lung cancer (Rajaee-Behbahani *et al*, 2001) and BC (Alapetite *et al*, 1999) have also been reported to exhibit the same range of DNA damage as control cells. Similarly, no difference has been revealed by the Comet assay between cells from control subjects and patients with *BRCA1* mutation, after irradiation with 2 Gy *in vitro* (Rothfuß *et al*, 2000).

In contrast to the above findings, increased basal as well as elevated radiation- and doxorubicin-induced DNA damage levels have been observed in the blood lymphocytes from BC patients (Blasiak *et al*, 2004; Colleu-Durel *et al*, 2004; Hussien *et al*, 2005).

Despite extensive studies into the relationship between cellular radiation tests, cancer risk and clinical radiation reaction, a convincing opinion has not been formed yet. The discrepancies cited above prompted us to explore whether the Comet and modified G2 MN assays are able to predict the clinical radiation reaction of BC patients and to discriminate them from healthy subjects. In addition, we analysed the influence of previous radiotherapy (RT) on the radiation response of cells *in vitro*. We examined both baseline and radiation-induced DNA damage in PBMCs from a much sizeable group of 50 unselected BC patients compared to that studied before (Djuzenova *et al*, 1999). PBMCs from a small group of cancer patients with an adverse early skin reaction to RT have also been included.

## MATERIALS AND METHODS

### Subjects

The assay was performed on PBMCs isolated from three groups of individuals: (1) a group (*n*=50) of unselected BC patients who were prospectively involved in the study and their blood samples were collected before clinical irradiation; (2) a group of cancer patients with an adverse early skin reaction (radiation therapy oncology group (RTOG) grades 2 and 3) to RT, who were retrospectively involved in the study, hereafter designated as ‘SC, sensitive cases’ and included seven patients with BC, one with tongue carcinoma (TC) and one with plasmacytoma (PC). Blood sampling was carried out during or after cessation of RT; and (3) a group of apparently healthy donors (*n*=16), mainly hospital personal and their relatives. To our knowledge, none of the healthy controls was previously exposed to radiation. All patients and healthy donors were asked to complete a questionnaire on their medical histories and lifestyles, including genetic diseases, medication, alcohol consumption, smoking, etc. Patients receiving chemotherapy prior to RT were excluded. The study was approved by the University of Würzburg Ethics Committee and all patients and donors gave informed consent.

Radiotherapy treatment of cancer patients was performed by means of a 6 MV linear accelerator (Siemens, Concord, CA, USA) at a dose rate of 2 Gy min^−1^. All BC patients received a tangential irradiation of the whole breast, with lateral and medial wedge fields. The regimen comprised a total dose of 50 or 60 Gy with a fractionation dose of 2 Gy five times a week. The early skin reaction to RT developing in the skin within the radiation field of the breast was used as an indicator for clinical radiosensitivity according RTOG score (Cox *et al*, 1995).

### Blood sampling and isolation of cells

PBMCs were separated from the heparinised blood samples by density-gradient centrifugation using Ficoll–Histopaque 1077 (Sigma 1077-1, Deisenhofen, Germany) according to the manufacturer's instructions. Peripheral blood mononuclear cells were washed twice with Ca^2+^- and Mg^2+^-free physiological phosphate-buffered saline (PBS; Sigma D-8537) and finally resuspended in the freezing medium containing RPMI 1640 (Sigma R-8758), 10% foetal calf serum and 10% dimethyl sulphoxide, and stored frozen in a liquid nitrogen until analysis.

### Alkaline Comet assay (slide method)

The Comet assay was performed under alkaline conditions following a modified protocol initially reported elsewhere (Singh *et al*, 1988; Djuzenova *et al*, 1999, 2001). Fully frosted microscope slides (SuperFrost Plus® Menzel GmbH, Braunschweig, Germany) were dipped up to one-fourth the frosted area in a hot 0.9% normal-melting-point agarose (Roth, 2268.2; Karlsruhe, Germany) in PBS. The slides were air-dried and stored at room temperature until needed. A measure of 80 *μ*l of cell/agarose suspension (10 *μ*l of cell suspension containing about 10^4^ cells was mixed with 70 *μ*l of 0.6% low-melting-point agarose, Roth, 6351.1, in PBS) was placed over the first agarose layer and allowed to solidify under a coverglass. After irradiation, coverglasses were removed, the slides were gently immersed in a cold lysis solution (2.5 M NaCl, 100 mM ethylenediaminetetraacetic acid (EDTA), 10 mM Tris, 1% sodium sarcosinate, pH was adjusted to 10 with NaOH, 10% dimethyl sulphoxide and 1% Triton X-100 added just before use) for 1 h at 4°C to lyse the cells and to permit DNA unfolding. The microscope slides were then transferred to the horizontal gel electrophoresis unit filled with fresh, chilled electrophoresis buffer (300 mM NaOH, 1 mM EDTA, pH 13.5) to a level of about 0.2 cm above the slides and left for 20 min to allow unwinding of DNA before electrophoresis. Electrophoresis was conducted for the next 20 min at 22 V (0.83 V cm^−1^). The slides were then drained, placed on a tray and flooded slowly with three changes of a neutralisation buffer (0.4 M Tris, pH 7.5), each for 5 min, to remove alkali. The slides were then stained with propidium iodide (Sigma P-4170, 10 *μ*g ml^−1^ solution in PBS), 50 *μ*l per slide, covered with a coverglass and placed in a humidified air-tight container to prevent drying of the gel, and were analysed within 24 h.

### *In vitro* X-ray irradiation for comet assay

At 2 h prior to irradiation, cells were thawed, centrifuged and resuspended in 1 ml of complete growth medium (CGM), consisting of RPMI 1640, supplemented with 10% foetal calf serum, 1% nonessential amino acids, 1 mM sodium pyruvate, 2 mM L-glutamine and penicillin/streptomycin (100 U ml^−1^ and 100 *μ*g ml^−1^, respectively). The final cell density of PBMCs was adjusted to 1 × 10^6^ cells ml^−1^ and the samples were placed at 37°C in a 5% CO_2_ incubator. X-irradiation was performed using a 6 MV Siemens linear accelerator (Siemens, Concord, CA, USA) at a dose rate of 2 Gy min^−1^. Cells embedded in agarose were irradiated on slides on crushed ice and were then placed in ice-cold lysis buffer (see below) or in CGM at 37°C in a water bath for specified times prior to lysis. Nonirradiated cells were treated in similar way (but at a zero radiation dose).

### Comet capture and analysis

Propidium iodide-stained electropherograms were examined in an epifluorescent microscope (Leica, DMLB, excitation filter: 515–560 nm; barrier filter: 590 nm) attached to a black and white CCD video camera (Cohu Electronics, San Diego, CA, USA), which was connected to a PC equipped with an image analysis software Komet 5.5. (Kinetic Imaging Ltd, Liverpool, UK). Between 75 and 100 randomly captured cells from each slide were analysed at each sampling time. Occasional dead cells, superimposed cells and cells on the edge of the gels were avoided.

Several features for each cell were evaluated by the software package, but the ‘tail moment’ (TM) was used here to quantitate the extent of DNA damage (Olive *et al*, 1990). The TM value (given in arbitrary units) is defined as the product of the percentage of DNA in the comet tail and the tail length. For each cell sample, the TM data were plotted *vs* time and fitted to a monoexponential function: 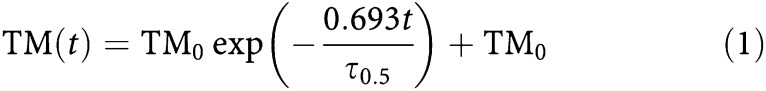 where *t* is the incubation time after X-ray exposure; TM_0_ and TM_R_ are the initial *repairable* TM and residual (i.e. irreparable) TM, respectively; and *τ*_0.5_ is the repair half-time, that is, the time required for cells to restitute 50% of the DNA damage. The *total* initial TM_I_ (*t*=0) is given by the sum: TM_I_=TM_0_+TM_R_.

### MN test

The MN assay was performed essentially according to Fenech (1993) with slight modifications. Peripheral blood mononuclear cells (2–5 × 10^6^ ml^−1^) were incubated in CGM containing 5 *μ*g ml^−1^ PHA (Sigma L-9132) for 2 days to stimulate the cell cycle progression. At 1 h before irradiation, cytochalasin B (5 *μ*g ml^−1^, Sigma C-6762) was added to prevent cytokinesis, which resulted in the appearance of binucleated cells (BNCs). After irradiation with a single dose of 1, 2, 3 and 4 Gy, the cells were cultured for additional 24 h, brought onto glass slides by cytospin centrifugation, fixed in cold methanol (−20°C, 1 h) and finally stained with acridine orange (62.5 *μ*g ml^−1^, Sigma A-4921) as described elsewhere (Stopper *et al*, 2005). The percentage of BNCs containing MN and the total numbers of MN per a single BNC were scored in 1000 BNCs using a × 400 magnification according the criteria published elsewhere (Fenech and Morley, 1985a). In the version of the MN test used here, PBMCs were at first stimulated with PHA (48 h) and then irradiated, so it was a G2 MN test as compared to the usually performed G0 MN test (Scott *et al*, 1998; Baeyens *et al*, 2002).

### Statistics

Data are presented as mean (±s.d.). Mean values were compared by the Student's *t*-test. The threshold of statistical significance was set at *P*<0.05. Statistics and fitting of experimental curves were performed with the program Origin 5.0 (Microcal, Northampton, MS, USA).

## RESULTS

### Comet assay

**Figure 1:**
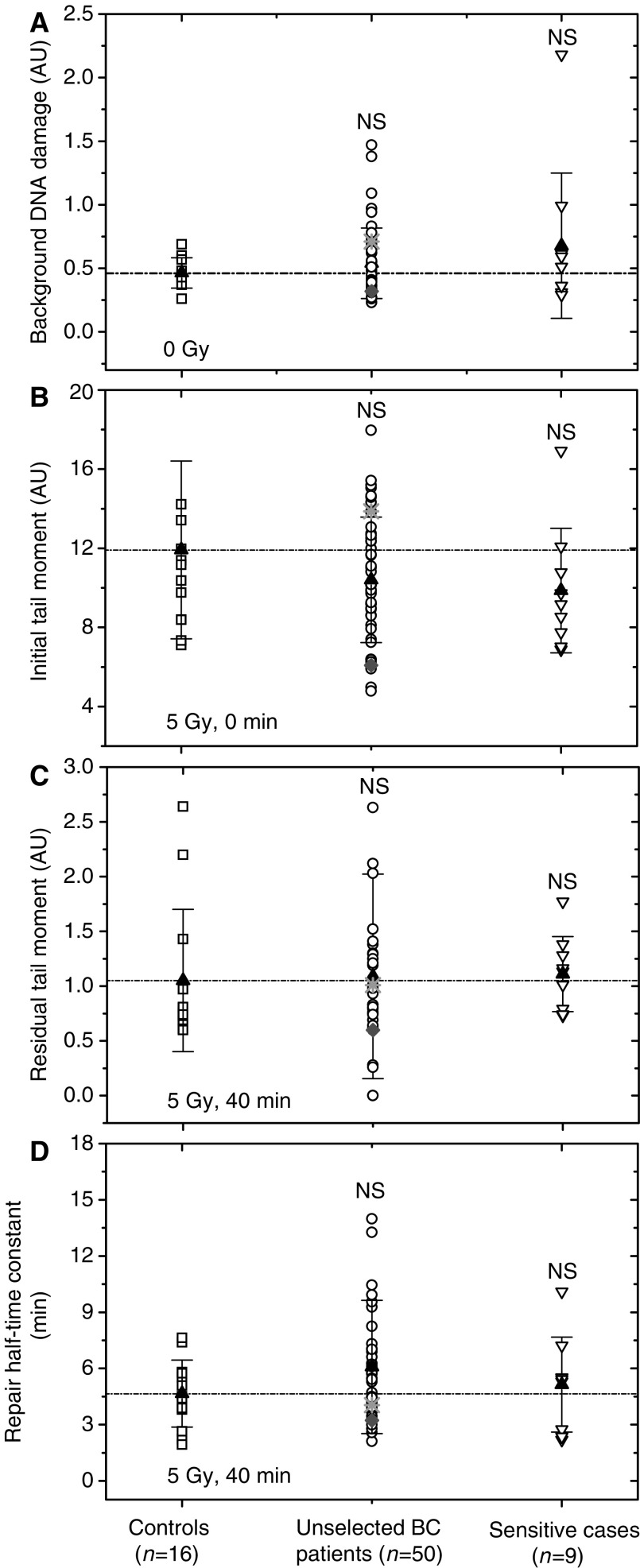
DNA damage assessed by means of the Comet assay in nonirradiated (**A**) as well as in irradiated (**B**–**D**) PBMCs derived from unselected BC patients (circles) and radiation-sensitive (unfilled down triangles) cancer patients compared with the cells from apparently healthy donors (squares). Initial (**B**), residual (**C**) DNA damage and the repair half-time constants (**D**) were assessed in PBMCs after irradiation with 5 Gy *in vitro*. Filled triangles represent the mean values for the respective group. ‘NS’ indicates that the difference was not highly significant (*P*>0.05). To facilitate visual comparison, the dashed line is drawn through the mean value of the group of healthy donors. Star and diamond represent the data for patients 016 and 021 who revealed an adverse skin reaction after RT.

The damage to DNA and its repair kinetics were evaluated up to 40 min after exposure to 5 Gy of X-rays, with a step of 10 min. The Comet TM (see Materials and Methods) was used to quantify the extent of DNA damage. The mean TM data were plotted as functions of time (data not shown) and fitted to equation (1) using the least-squares method. From the best fits, the values of the initial (TM_I_) and residual (TM_R_) tail moment, as well as the repair half-time (*τ*_0.5_) were determined for the cell samples from each tested individual (Figure 1).

The fitted parameters of DNA damage repair curves for each individual, as well as the age, sex, clinical and smoking status, are given in Table 1, from which the following trends are obvious (see also Figure 1). Although nonirradiated cells of some cancer patients exhibited noticeably higher baseline amounts of DNA fragmentation, the *mean* values of background DNA damage were similar in the three tested groups of individuals (Figure 1A). Likewise, after irradiation *in vitro*, the Comet assay did not reveal any differences among the three groups in terms of their initial (Figure 1B) and residual levels of DNA damage (Figure 1C) as well as in the DNA repair kinetics (Figure 1D), which may be in part due to the strong interindividual variability.

Out of 50 prospectively recruited BC patients, only two (016 and 021) exhibited an increased early skin reaction to RT (both grades 2 and 3 according RTOG score) so the group of ‘unselected BC’ patients could be also viewed as normally reacting patients. However, only for one of them (Figure 1B, star), the increased clinical radiosensitivity, correlated with a somewhat higher initial DNA damage level detected by the Comet assay. Other parameters of the Comet assay on the cells from these two patients did not deviate significantly from controls.

### MN test

**Figure 2:**
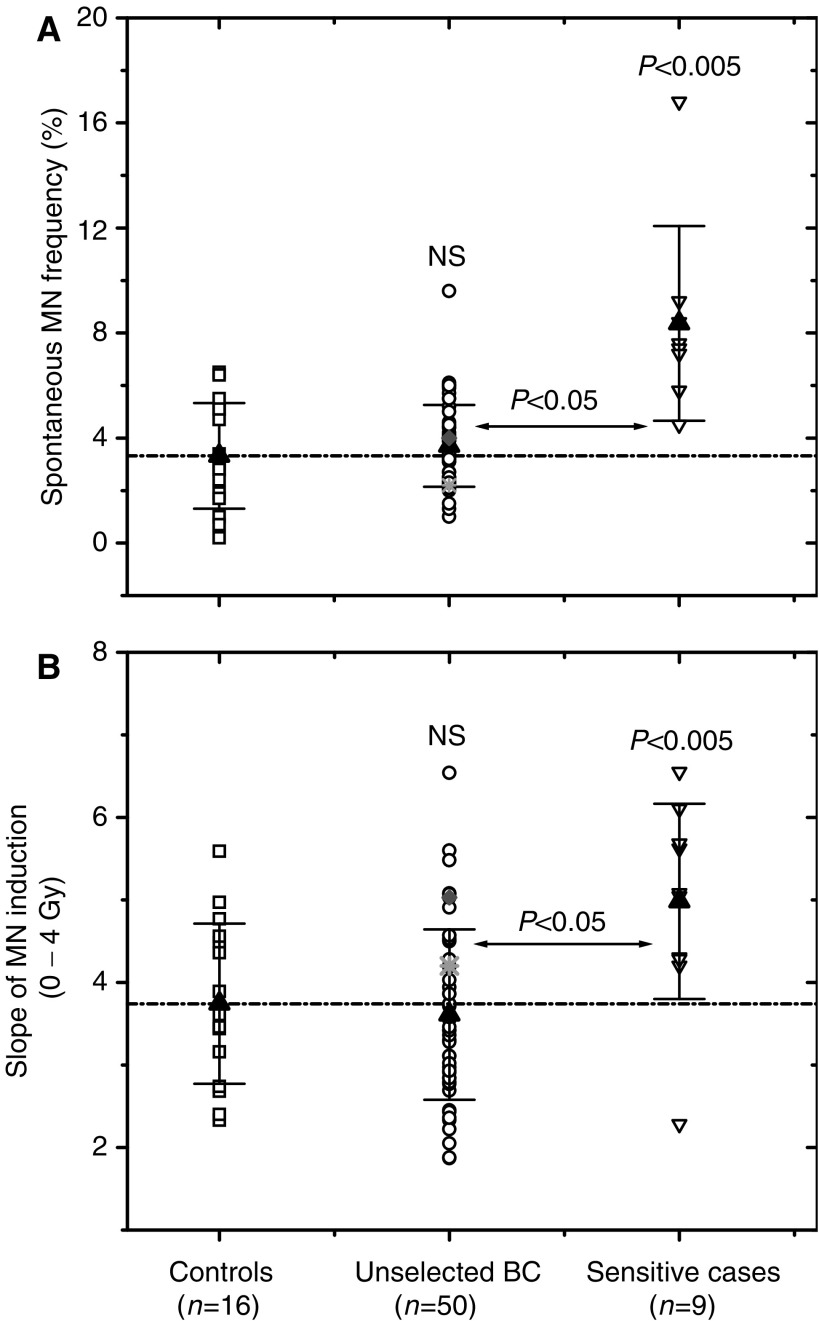
Spontaneous MN expression (**A**) and the slopes (**B**) of the dose–response curves for MN induction after *in vitro* irradiation with 0–4 Gy in PBMCs derived from unselected (circles) and sensitive (unfilled down triangles) cancer patients are shown in comparison with those in the cells from control subjects (squares). Each data point in (**A**) represents the mean frequency of micronucleated BNCs scored in 1000 BNCs. Each data point in (**B**) represents the slope of the dose–response curve for MN induction in 1000 BNCs (per a single dose of radiation) from a given individual. Filled triangles represent the mean values for the respective group. ‘NS’ indicates that the difference was not highly significant (*P*>0.05). To facilitate visual comparison, the dashed line is drawn through the mean value of the group of healthy donors. Star and diamond represent the data for patients 016 and 021 who revealed an adverse skin reaction after RT.

**Figure 3:**
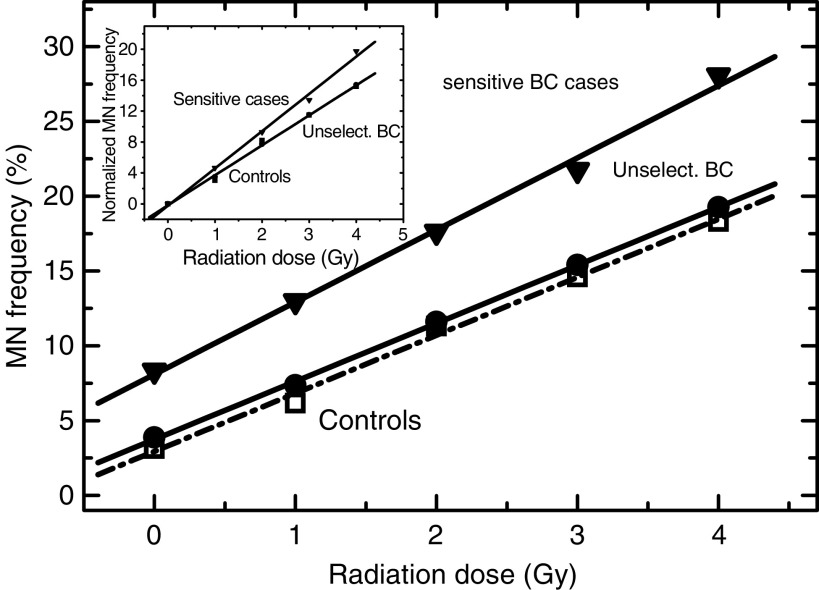
Spontaneous and radiation-induced MN expression in PBMCs averaged through the group of healthy control donors (squares and dashed line), unselected BC patients (filled circles) and cancer patients with adverse skin reaction to RT (‘SCs’, down triangles). Inset shows the dose–response curves normalised by subtracting the respective baseline MN expression in nonirradiated cells.

In addition to the Comet assay, the cell samples were analysed by a cytochalasin-blocked MN test (Fenech and Morley, 1985a) with a minor modification concerning mitogen stimulation. This test represents a well-established assay for biomonitoring human populations as an indicator of genetic instability and radiation damage (Müller *et al*, 2003). Using the G2 MN test, both percentage of BNCs with MN (Figures 2, 3, 5) and total numbers of MN per single BNC (not shown) were scored in a total number of 1000 BNCs. As seen from Figure 2A, there was no difference in the spontaneous MN yields between unselected BC patients and healthy controls. In contrast, the PBMCs from cancer patients with an adverse skin reaction to RT exhibited a significantly higher amounts of spontaneous MN compared to both control and unselected BC patients (Figure 2A; *P*<0.005 and *P*<0.05, respectively).

In addition to the spontaneous MN formation (Figure 2A), the induction of MN upon irradiation *in vitro* with 0–4 Gy was evaluated and the slopes of the dose–response curves were analysed for each group (Figure 2B). As with the spontaneous MN levels (Figure 2A), the mean slope of MN induction in cells from cancer patients with an adverse skin reaction was significantly higher than in both control and unselected BC group. The latter two groups exhibited similar dose–response slopes in the G2 MN test.

In the case of two BC patients (016 and 021) who developed an increased early skin reaction to RT (Figure 2A, star and diamond), the mean spontaneous MN yields in their blood cells were similar to that in control. However, after *in vitro* irradiation, cells derived from one of them (patient 021) showed a somewhat higher rate of MN induction (Figure 2B, diamond).

As an extension of Figure 2B, Figure 3 illustrates the radiation dose dependences of MN induction averaged through each tested group of individuals. The dose–response curve of MN induction in cells from unselected BC patients (Figure 3, circles) is similar to that obtained for control cells (Figure 3, squares). On the other hand, cells from cancer patients with an adverse skin reaction to RT (Figure 3, ‘SC’, triangles) showed significantly higher MN induction levels compared to controls and unselected BC patients. As there was an increased amount of spontaneous MN in cells from radiation-sensitive patients (see also Figure 2A), we additionally normalised the dose–response data by subtracting the background MN levels (Figure 3, inset). In the normalised plot, cells from radiation-sensitive cancer patients exhibited the highest relative rate of MN induction *in vitro*, whereas the dose–response of cells from unselected BC patients was similar to that of control group.

### Effects of previous RT on the radiation response of patients' cells *in vitro*

**Figure 4:**
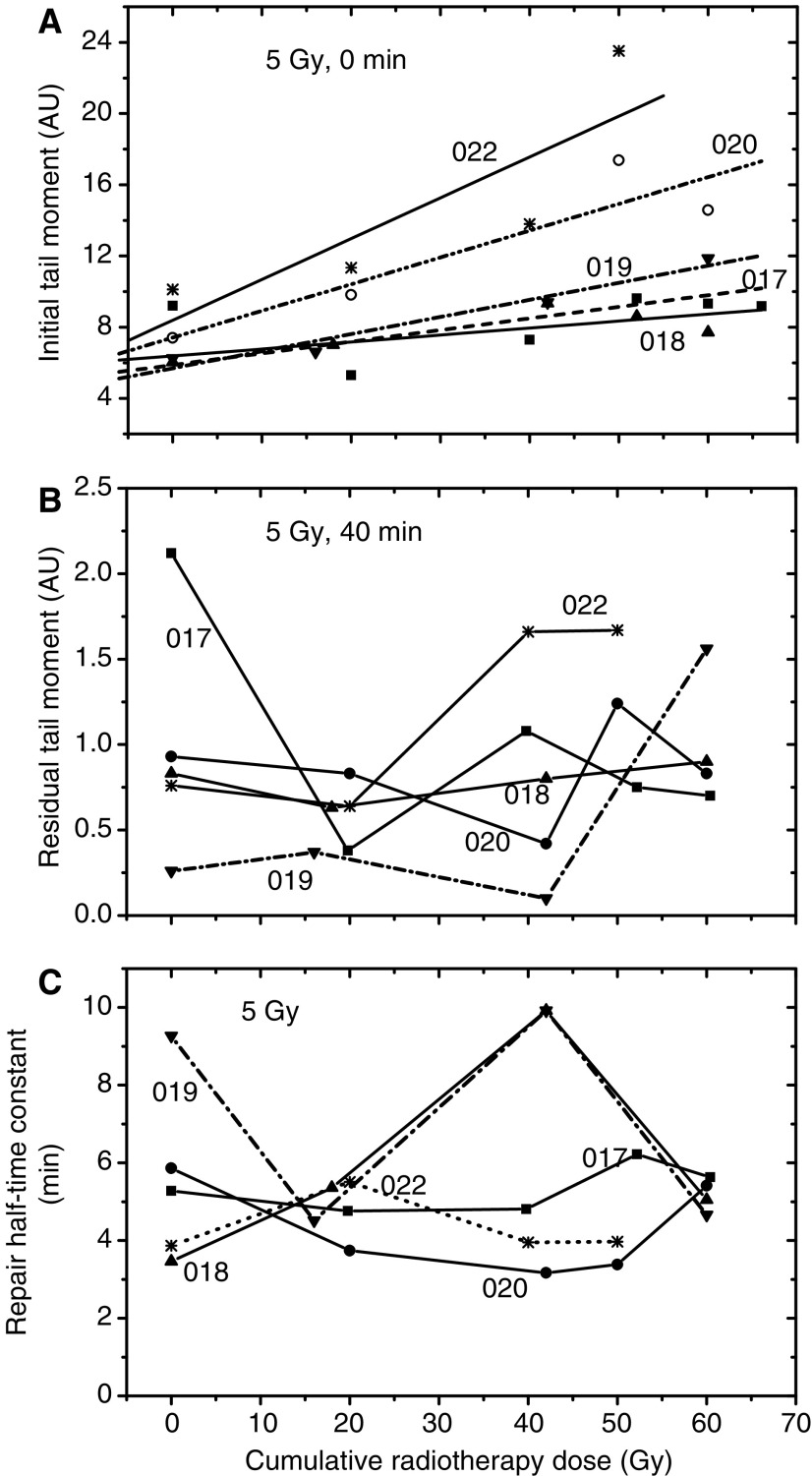
Effects of foregoing RT on the radiation response *in vitro* of cells from five breast cancer patients (017, 018, 019, 020 and 022, *see* Table 1) with normal clinical radiosensitivity assessed by the Comet assay. (**A**–**C**) The initial and the residual DNA damage, and the repair half-time constants, respectively. Data were averaged through 75 cells from a given individual per dose and time point. Blood samples were collected before, during (20 and 40 Gy) and after cessation (50–60 Gy) of RT. Bars for the means have been omitted for clarity.

As mentioned above, blood samples of cancer patients with an adverse skin reaction to RT (group 2 in Materials and Methods) were collected during or after clinical irradiation. In order to investigate the influence of previous RT on the radiation response of patients' cells *in vitro*, blood samples from five unselected BC patients were taken before, during (at 20 and 40 Gy) and after (50–60 Gy of cumulative RT dose) clinical irradiation and analysed by the Comet and MN assays. The Comet assay applied after 5 Gy *in vitro* revealed that the initial DNA fragmentation rose with increasing cumulative dose of RT in cells of four out of five tested patients (Figure 4A). Unlike the initial DNA damage, no conclusions could be drawn about the influence of previous RT on the residual DNA damage and repair half-time (Figure 4B and C, respectively), mainly because of the wide individual variability of these parameters. In addition, the background DNA damage level (i.e. tail moment at 0 Gy *in vitro*) remained unchanged after clinical irradiation (data not shown).

**Figure 5:**
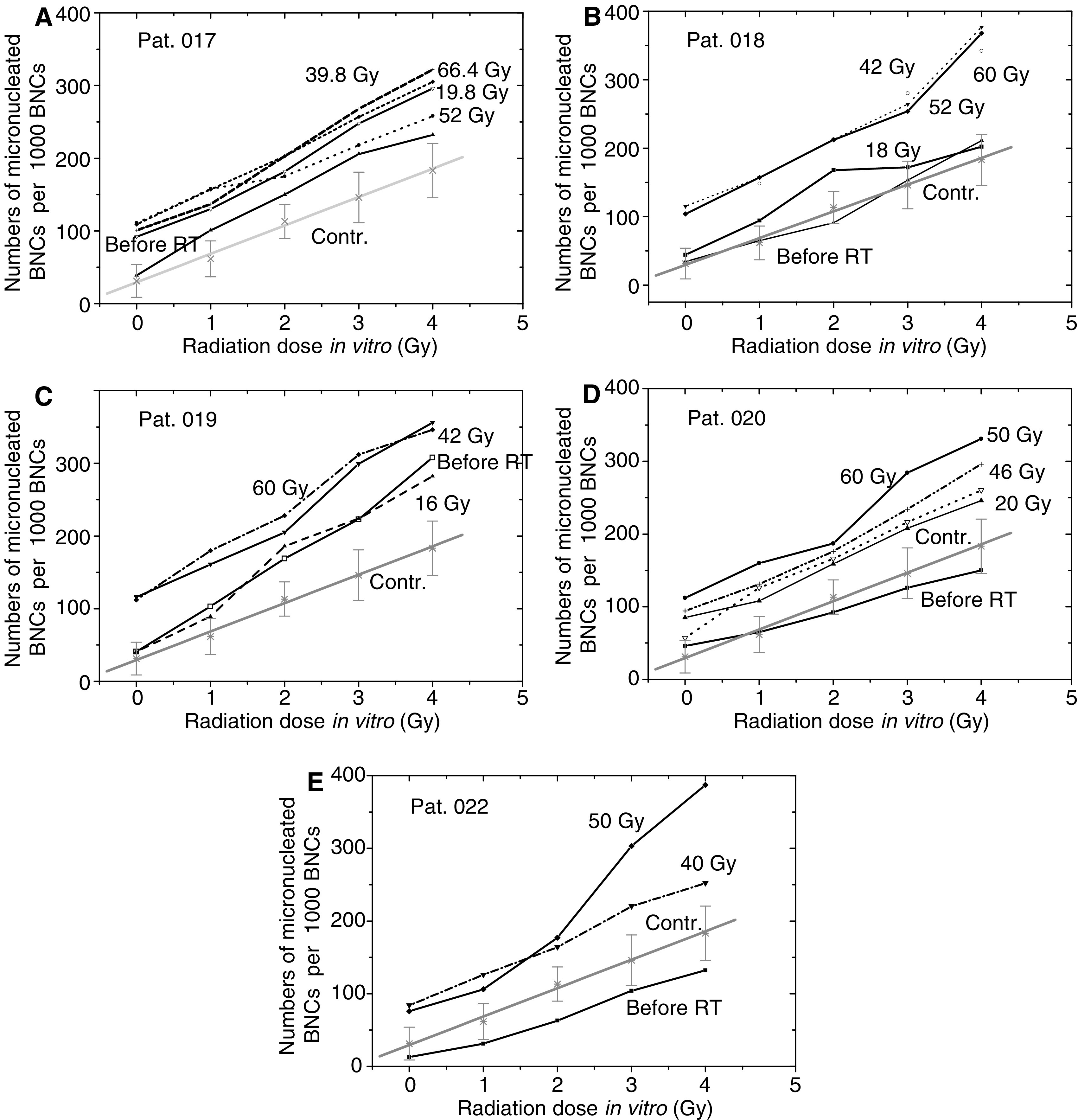
Effects of foregoing RT on the MN induction *in vitro* in cells from five breast cancer patients with normal clinical radiosensitivity. Blood samples were collected before, during (20 and 40 Gy) and after cessation (50–60 Gy) of RT. Each symbol represents the mean numbers of BNCs containing MN scored in 1000 BNCs. Bars for the means have been omitted for clarity. For comparison, the mean (±s.d.) dose–response curve (stars) for the group of healthy donors is depicted in each part.

The G2 MN assay of the same cell probes shown in Figure 4 revealed that previous RT exerted strong effects on both baseline and *in vitro* radiation induced MN yields (Figure 5). Thus, for a given radiation dose *in vitro* (0–4 Gy), the mean MN frequencies grew steadily with increasing cumulative RT dose for all five tested BC patients (Figure 5A–E).

## DISCUSSION

In this study, peripheral blood cells isolated from (1) unselected BC patients, (2) cancer patients with an adverse early skin reaction to RT, and (3) healthy individuals were analysed for their DNA fragmentation using the Comet assay. The analysis of both nonirradiated and *in vitro*-irradiated cell samples did not reveal any differences in the background or radiation-induced DNA damage levels among the three groups. There was a trend toward an increased background level in the cells from both groups of cancer patients, but this failed to reach statistical significance. The Comet data obtained here are in agreement with the findings that the baseline DNA damage levels in nonirradiated peripheral blood lymphocytes from patients with multiple tumours (Müller-Vogt *et al*, 2003), lung cancer (Rajaee-Behbahani *et al*, 2001) and breast cancer (Alapetite *et al*, 1999) are similar to healthy controls. Our results, although without accounting for *BRCA1* and *BRCA2* mutations, but on a sizeable sample (*n*=50) of unselected BC patients, are also in line with the finding of Rothfuß *et al* (2000) that the Comet assay has not revealed any difference between the *in vitro*-irradiated cells from four patients with *BRCA1* mutation and those from four control subjects.

On the other hand, our results disagree with those of Colleu-Durel *et al* (2004) and Hussien *et al* (2005), who have found increased levels of both basal and radiation-induced DNA damage in cells from BC patients, as compared to healthy controls. The reasons for the discrepancy might reside in the patients' and controls' cohorts, cancer stage, treatment prior to blood sampling, arbitrary determined cut off values, experimental protocols as well as in interlaboratory variability. Thus, in contrast to the present and several other studies (Alapetite *et al*, 1999; Rothfuß *et al*, 2000; Müller-Vogt *et al*, 2003), in which healthy individuals were used as references, the control group in Hussien *et al* (2005) includes patients with the benign breast disease. Besides this, even if the same version of the alkaline Comet assay (Singh *et al*, 1988) was used here and elsewhere (Colleu-Durel *et al*, 2004; Hussien *et al*, 2005), the quantitative TM data appear to differ greatly between laboratories. Thus, the TM values of 7.74 and 14.67 in nonirradiated cells from control and BC patients, respectively, obtained by Colleu-Durel *et al* (2004) are larger some 20–30-fold than the TM values of 0.47 and 0.45 presented here in Figure 1A. For comparison, the TM values of 1.4 and 1.21 have been found, respectively, for bladder cancer patients and controls (Schabath *et al*, 2003), whereas TM values of about three have been obtained for both breast cancer patients and controls by Alapetite *et al* (1999).

The second screening test used here was the MN assay. Unlike the usual version of the MN test, which involves irradiation of quiescent G0 cells followed by mitogen stimulation (Scott *et al*, 1998; Burrill *et al*, 2000; Baeyens *et al*, 2002), in the present study, the cells were stimulated with PHA for 48 h prior to irradiation, as commonly used for assessing G2 chromosomal aberrations (Scott *et al*, 1998). We found that the mean frequencies of both spontaneous and radiation-induced MN in PBMCs from unselected BC patients were similar to those in control. Consistent with the G2 MN data presented here, the G0 MN assay also has failed to discriminate a group of cervical, head and neck cancer patients from healthy individuals (Slonina *et al*, 2000). In addition, the radiomimetic drug bleomycin induces similar rates of chromatid breaks in lymphocytes from BC patients and from healthy controls (Hsu *et al*, 1989).

On the other hand, the data for the group of unselected BC patients obtained here using the G2 MN test seem to disagree with those from several studies (Scott *et al*, 1998; Burrill *et al*, 2000; Baeyens *et al*, 2002), in which the G0 MN test has detected increased radiosensitivity in cells from BC patients compared to healthy controls (reviewed in Speit and Trenz, 2004). The apparent conflict, however, can be explained by differences in both experimental and statistical approaches used here and elsewhere (Scott *et al*, 1998; Burrill *et al*, 2000; Baeyens *et al*, 2002). Firstly, we used a modified G2 version of the MN test instead of the classic G0 MN assay. Secondly, we compared the mean values of groups rather than the data ranges. Thirdly, we did not set any arbitrary cut off values. Finally, the control and patients' cohorts differ markedly among studies.

We also found that blood cells from cancer patients with an adverse early skin reaction to RT displayed increased frequencies of both spontaneous and radiation-induced MN compared to the cells from apparently healthy donors and unselected BC patients (Figures 2 and 3). This result is in agreement with the findings that MN levels are elevated in cells from BC patients with known genetic predisposition (Rothfuß *et al*, 2000; Baeyens *et al*, 2002). It has to be noted, however, that in our study the radiation-sensitive cancer patients were recruited based on the knowledge of their clinical reaction to RT, so that blood sampling was performed during or after cessation of RT. This fact raised an important question whether RT itself affects the outcome of *in vitro* tests. To check this possibility, we further analysed blood samples (of five patients) collected prior, during (20 and 40 Gy) and after (50–60 Gy) cessation of RT. A striking effect of the radiation *in vivo* on the spontaneous and radiation-induced MN yields *in vitro* was observed in all five tested patients (Figure 5). These data suggest that clinical radiation might have provoked genetic instability and/or induced persistent (nonrepaired) DNA damage in the normal cells of cancer patients, thus leading to increased levels of MN induction and DNA fragmentation after irradiation *in vitro*. Our data agree with the observations that MN levels rise with increasing cumulative RT dose in cancer patients (Fenech *et al*, 1990; Cao *et al*, 2002), although some studies report a lack of difference in the G2 chromosomal radiosensitivity between pre- and post-therapy samples (Roberts *et al*, 1999; Baeyens *et al*, 2002). Furthermore, the increased frequency of *spontaneous* MN in cells from BC patients has been suggested to be caused, at least partly, by previous radio- and/or chemotherapy (Baeyens *et al*, 2002), which is in agreement with our data presented in Figure 5.

Unlike the MN test, the Comet assay revealed only a weak correlation between the initial DNA damage and cumulative RT doses (Figure 4A), whereas other parameters, including the residual DNA damage and repair half-time constants, do not allow any conclusions to be made, mainly because of the wide data scattering. In addition, the Comet assay did not detect any changes in the background DNA fragmentation in peripheral lymphocytes from BC patients subjected to RT (data not shown).

It may be argued that in the present study the small control group (an average age of 41±18 years) was much younger than each tested patients' group (mean age of 61±10 and 55±9). However, it has been shown earlier that while the basal levels of DNA damage in the Comet assay are independent of the age of the donor, an age-dependent increase in DNA damage has been observed immediately after irradiation (Singh *et al*, 1990). In addition, a nearly four-fold increase in MN in cultures from 80-year-old donors was measured compared with that in the cultures from newborn donors (Fenech and Morley, 1985b), and an age dependent increase of 0.58 MN year^−1^ was found for a female population (Thierens *et al*, 2000). Taking these data into account, the cells from the much elderly group of BC patients studied here could be expected to exhibit increased levels of both radiation-induced DNA damage and spontaneous MN expression, as compared with those from the younger controls. However, at variance with expectation, there was no significant difference in the induced DNA damage between the group of unselected BC patients and controls (Figures 1 and 2). Likewise, the two groups exhibited similar baseline frequencies of MN expression (Figure 2A). Another point is that our control group includes several males, whereas BC patients' groups include only females. However, the effect of gender on the outcome of both Comet and MN tests has been reported to be negligible (Garaj-Vrhovac and Kopjar 2003; Hadjidekova *et al*, 2003).

As fortunately a minority (about 5%) of RT patients develop during or after RT either acute or late radiotoxic responses (Norman *et al*, 1988), a cohort of 50 prospectively recruited BC patients in our study was too small to find several radiation-sensitive patients. Indeed, we observed only two patients exhibiting early skin radiotoxicity during or shortly after RT. Even so, there was a weak correlation of radiation response *in vivo* with either an increased DNA damage level in Comet assay or an increased MN induction in one or another case. Furthermore, the present study is ongoing, so that potential late effects of RT are still to be analysed and they will be the subject of future follow-up.

In conclusion, neither prospectively recruited BC patients nor sensitive cancer patients with an adverse skin reaction to RT could be identified by the Comet assay. In contrast, the modified G2 MN test detected significantly increased levels of both spontaneous and radiation-induced MN in cells from sensitive cancer patients. However, the G2 MN assay was unable to identify normally reacting BC patients. The increased MN rates in sensitive cancer patients might have been in part a consequence of the previous RT, which was found to affect strongly the outcome of the MN test *in vitro*. A larger study is required to investigate to what extent RT alters the radiation response of patients' cells *in vitro*.

## Figures and Tables

**Table 1:** DNA damage measured by the Comet assay in PBMCs isolated from blood of apparently healthy donors (*N*), unselected BC patients and cancer patients with adverse skin reaction to RT after exposure to 5 Gy of X-irradiation *in vitro*^a^

**Subject^b^**	**Age (years)**	**Sex**	**Smoking status^c^**	**Clinical status with respect to cancer, skin reaction to RT^d^**	**0 Gy, TM (AU)**	**Total initial TM, TM_IT_ (AU)**	**Residual TM, TM_R_ (AU)**	**Repair half-time (min)**
*Apparently healthy donors*								
N-1	26	F	—	ND	0.44	7.11	0.69	5.81
N-2	27	F	—	ND	0.38	11.39	2.20	2.23
N-3	39	M	—	ND	0.60	11.97	0.76	4.51
N-4	30	F	—	ND	0.48	7.34	0.80	7.39
N-5	51	F	—	ND	0.57	10.37	0.61	3.80
N-6	32	M	10/day	ND	0.69	11.17	0.81	4.40
N-7	31	M	—	ND	0.37	9.76	0.97	2.64
N-8	25	M	—	ND	0.44	13.42	0.60	5.74
N-9	24	M	—	ND	0.26	14.22	0.73	5.08
N-10	27	F	—	ND	0.45	8.40	0.74	3.89
N-11	25	F	—	ND	0.43	8.38	1.43	7.63
N-12	62	F	—	ND	0.74	9.75	1.27	4.26
N-13	49	F	—	ND	0.48	11.06	1.10	4.84
N-14	67	F	—	ND	0.36	10.20	1.10	0.48
N-15	66	F	—	ND	0.61	10.05	0.63	6.05
N-16	77	F	—	ND	0.59	10.0	1.67	4.78
Mean	41				0.49	10.30	1.0	4.60
±s.d.	18				0.13	2.0	0.45	1.82
								
*BC patients*								
001	62	F	5–10/day	BC, grade 2	0.62	9.82	1.38	4.30
002	63	F	—	BC, grade 2	1.09	12.34	1.41	6.89
003	68	F	—	BC, grade 1	0.97	8.61	0.79	4.71
004	65	F	—	BC, grade 1	0.42	12.74	1.00	5.70
005	58	F	—	BC, grade 2	0.36	12.52	1.07	4.41
006	79	F	—	BC, grade 1	0.27	9.71	0.62	4.50
008	64	F	—	BC, grade 1	0.39	15.05	0.69	6.27
009	47	F	10/day	BC, grade 1	0.29	10.14	0.75	10.45
010	75	F	—	BC, grade 1	0.39	11.01	0.10	13.27
012	58	F	—	BC, grade 2	0.83	17.96	1.03	3.67
013	55	F	—	BC, grade 1	0.42	11.99	1.30	4.46
014	73	F	—	BC, grade 1	0.48	10.81	1.29	2.11
015	72	F	—	BC, grade 1	0.55	6.36	0.83	7.04
**016**	**66**	**F**	—	**BC, grades 2 and 3**	**0.71**	**13.87**	**1.01**	**4.04**
017	56	F	—	BC, grade 1	0.23	9.18	0.80	5.28
018	55	F	10–15/day	BC, grade 0 and 1	0.36	6.03	0.83	3.46
019	69	F	—	BC, grade 0 and 1	0.50	6.25	0.26	9.27
020	75	F	—	BC, grade 0 and 1	0.45	7.40	0.93	5.86
**021**	**69**	**F**	—	**BC, grades 2 and 3**	**0.32**	**6.08**	**0.60**	**3.23**
022	62	F	—	BC, grade 1	0.27	10.13	0.76	3.87
023	63	F	—	BC, grade 1	0.45	8.92	1.19	6.60
024	70	F	—	BC, grade 1	0.34	7.25	1.25	2.77
025	54	F	2–5/day	BC, grade 1	0.33	5.90	0.81	4.41
026	74	F	—	BC, grade 0	0.26	4.78	0.74	4.13
027	38	F	—	BC, grade 2	0.55	12.36	0.64	8.25
028	59	F	—	BC, grade 1	0.94	13.81	1.52	9.56
029	67	F	—	BC, grade 2	0.45	9.89	0.98	5.75
030	63	F	—	BC, grade 1	0.78	12.65	1.21	4.21
031	47	F	15–20/day	BC, grade 1	0.69	14.58	2.63	4.20
032	68	F	—	BC, grade 0	0.73	14.65	2.37	3.29
033	70	F	—	BC, grade 1	0.56	8.10	0.66	9.93
034	75	F	—	BC, grade 1	0.78	8.59	1.0	7.02
035	54	F	—	BC, grade 1	0.41	11.68	1.11	4.45
036	48	F	—	BC, grade 1	0.31	9.27	1.07	3.17
037	58	F	—	BC, grade 1	0.34	6.26	0.65	4.09
038	75	F	2–5/day	BC, grade 1	0.37	11.21	1.32	3.38
039	60	F	—	BC, grade 2	0.47	13.26	1.21	4.30
040	54	F	—	BC, grade 2	0.67	12.7	1.45	4.52
041	45	F	—	BC, grade 1	0.41	12.53	1.35	4.40
042	57	F	—	BC, grade 1	0.66	10.61	0.88	6.33
043	41	F	—	BC, grade 2	0.33	8.05	0.89	3.76
044	67	F	—	BC, grade 1	0.65	11.1	0.87	2.99
045	61	F	—	BC, grades 0 and 1	0.31	13.07	1.09	6.22
046	69	F	—	BC, grade 1	0.27	9.19	0.43	7.31
047	55	F	6–8/day	BC, grade 2	0.34	7.93	0.48	6.10
048	61	F	—	BC, grade 2	0.64	15.18	1.52	7.02
049	71	F	—	BC, grade 1	0.51	10.17	0.81	5.47
050	60	F	20/day	BC, grade 1	0.40	8.96	1.13	3.94
051	43	F	2–3/day	BC, grade 2	0.39	9.26	1.02	3.26
052	56	F	—	BC, grade 1	0.31	15.43	1.02	5.42
Mean	61				0.49	10.50	1.02	5.38
±s.d.	10				0.20	3.0	0.43	2.23
						**NS**	**NS**	**NS**
*Cancer patients with adverse skin reaction to RT*								
SC1	43	F	—	BC, grades 2 and 3	0.29	8.52	0.73	7.20
SC2	50	M	—	TC, grades 2 and 3	2.18	9.71	1.77	5.51
SC3	49	F	5–10/day	BC, grades 2 and 3	1.0	9.15	0.79	10.09
SC4	49	M	—	BC, grades 2 and 3	0.51	7.74	1.13	5.29
SC5	64	M	—	PC, grades 2 and 3	0.31	10.77	1.16	2.17
SC6	52	F	2–5/day	BC, grade 3	0.61	6.87	1.01	5.50
SC7	49	F	—	BC, grades 2 and 3	0.29	12.08	0.74	5.40
SC8	63	F	—	BC, grades 2 and 3	0.59	7.02	1.28	2.74
SC9	69	F	—	BC, grades 2 and 3	0.36	16.92	1.38	2.35
Mean	54				0.68	9.9	1.1	5.14
±s.d.	9				0.61	3.2	0.3	2.53

BC=breast cancer; F=female; M=male; N=normal; ND=not determined; PBMC=peripheral blood mononuclear cells; PC=plasmacytoma; RT=radiotherapy; SC=sensitive case; TC=tongue carcinoma.

a Each indicated DNA damage parameter represents the mean value obtained on the cells from a given individual. Significance was established using Student's *t*-test. ***P*** compares the differences between tested group and apparently healthy individuals. **NS** indicates that the difference was not highly significant (*P*>0.05). Bold fonts indicate the unselected BC patients who revealed an adverse skin reaction to RT.

b Case number was according to our files.

c ‘—’ means nonsmoker, the numbers indicate the amounts of smoked cigarettes per day.

d Early skin reaction according RTOG score (Cox *et al*, 1995). RTOG grade: 1 – follicular, faint or dull erythema, dry desquamation; 2 – tender or bright erythema, moderate oedema; 3 – confluent, moist desquamation, pitting oedema; 4 – ulceration, haemorrhage, necrosis.
